# Adipose tissue‐derived mesenchymal stem cells' acellular product extracellular vesicles as a potential therapy for Crohn's disease

**DOI:** 10.1002/jcp.30756

**Published:** 2022-05-06

**Authors:** Jessica Altemus, Neda Dadgar, Yan Li, Amy L. Lightner

**Affiliations:** ^1^ Department of Colorectal Surgery, Digestive Disease Surgical Institute Cleveland Clinic Cleveland Ohio USA; ^2^ Department of Inflammation and Immunity, Lerner Research Institute Cleveland Clinic Cleveland Ohio USA

**Keywords:** Crohn's disease, extracellular vesicles, M1/M2 macrophage polarization, mesenchymal stem cells

## Abstract

The breakdown of gastrointestinal tract immune homeostasis leads to Crohn's disease (CD). Mesenchymal stem cells (MSCs) have demonstrated clinical efficacy in treating CD in clinical trials, but there is little known about the mechanism of healing. Considering the critical roles of macrophage polarization in CD and immunomodulatory properties of MSCs, we sought to decipher the interaction between adipose‐derived MSCs and macrophages, including their cytokine production, regulation of differentiation, and pro‐/anti‐inflammatory function. RNA extraction and next generation sequencing was performed in adipose tissue from healthy control patients' mesentery (*n* = 3) and CD mesentery (*n* = 3). Infiltrated macrophage activation in the CD mesentery was tested, MSCs and extracellular vesicles (EVs) were isolated to compare the regulation of macrophage differentiation, cytokines production, and self‐renewal capacities in vitro. CD patients' mesentery has increased M1 macrophage polarization and elevated activation. MSCs and their derived EVs, isolated from inflamed Crohn's mesentery, leads to a rapid differentiation of monocytes to a M1‐like polarized phenotype. Conversely, MSCs and their derived EVs from healthy, non‐Crohn's patients results in monocyte polarization into a M2 phenotype; this is seen regardless of the adipose source of MSCs (subcutaneous fat, omentum, normal mesentery). EVs derived from MSCs have the ability to regulate macrophage differentiation. Healthy MSCs and their associated EVs have the ability to drive monocytes to a M2 subset, effectively reversing an inflammatory phenotype. This mechanism supports why MSCs may be an effective therapeutic in CD and highlights EVs as a novel therapeutic for further exploration.

## Graphical abstract

Chron's diseased mesenteric mesnechymal stem cells and associated extracellular vessicals polarize macrophages to an inflammatory M1 phenotye.

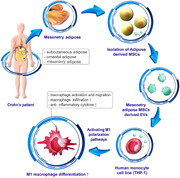

## 1 INTRODUCTION

Tissue damage provides a need for the host to neutralize invasive microorganisms, clear the damaged cell and debris sites, and then restore and rebuild the tissue (Faz‐Lopez et al., 2016). Therefore, the initiation of an inflammatory defensive reaction has developed to facilitate leukocyte and macrophage migration from the systemic circulation to local damaged tissue, removal of invasive pathogenic agents, and initiate tissue repair and functional recovery (Laskin et al., 2011; Wynn & Vannella, 2016). Ideally, the inflammatory response is an independent mechanism leading to complete resolution of inflammation and a rapid return to tissue homeostasis (Schett & Neurath, 2018; Sugimoto et al., 2016). In recent decades, inflammation resolution has been considered a passive operation; unique molecular and cellular mechanisms involved in inflammation resolution have been identified in which macrophages play an important role in preventing excessive immune responses (Chen et al., 2018; Serhan et al., 2007; Sugimoto et al., 2016). Indeed, after damage to tissue, phagocytosis of apoptotic cells (efferocytosis) causes a functional transition to an anti‐inflammatory transcript program, which is close to the alternative macrophage activation pattern, with cytokine production, growth factors, and lipid mediators necessary for the reintroduction of homeostasis (Arienti et al., 2019; Fadeel et al., 2010).

In the healthy intestinal lamina propria, macrophages are the most abundant mononuclear phagocyte to facilitate essential immune functions (Wang et al., 2019). Following the microenvironment changes, these macrophages are polarized into two classic macrophage phenotypes, a proinflammatory (M1) and anti‐inflammatory macrophage (M2) populations (Atri et al., 2018). During an inflammatory response, both phenotypes may turn into one another. The M1 and M2 cells are believed to be able to conduct double‐handed activities either by inducing inflammatory effects to enhance inflammation or by anti‐inflammatory functions to facilitate healing (Alvarez & Liu, 2016). Therefore, the immune system responds according to the macrophage phenotype, with or without inflammatory or noninflammatory cytokines, which is highly necessary for inflammation progression and forecast (Labonte et al., 2014). Under normal conditions, inflammatory pathways inhibit immunopathogenesis. Thus, imbalances between M1 and M2 switching are key problems that cause a number of disorders like Crohn's disease (CD) (Lee et al., 2018). In addition to preventing unnecessary inflammation of harmless commensal microorganisms, intestinal macrophages foster tolerance by favoring the expansion of mesenchymal stem cells (MSCs), particularly by developing their anti‐inflammatory and regenerative function. MSC‐based treatment is intended to resolve CD by adjusting the immune response to restore equilibrium and repair intestinal tissue damage including immune cell balance and microbiome diversity. Based on this data, a new perspective on the pathogenesis of CD and treatment methods could be given by understanding the function of macrophages and MSCs in the intestinal inflammatory response in CD specifically.

## 2 METHODS

### 2.1 Institutional review board

This study was approved by and performed in accordance with the Cleveland Clinic's Institutional Review Board (IRB# 19‐908). The colorectal surgery biobank is a patient identified repository for inflammatory bowel disease (IBD), colorectal cancer, and healthy control patients (i.e., hernia, diverticulitis, and functional bowel disorders). At the time of operative intervention, surgical specimen tissue, subcutaneous adipose tissue, mesenteric adipose tissue, omental tissue, blood, and stool may be collected. Adult patients with preoperative consent are included. Exclusion criteria include patients under the age of 18, pregnant women, lactating women, and individuals unable to provide informed consent. Patient and disease‐related variables are entered into a prospectively maintained database with REDCap.

### 2.2 RNA extraction and next generation sequencing (NGS)

RNA was isolated from the mesentery (Mes) and subcutaneous (SubQ) adipose tissue of three patients with diverticulitis (Healthy control) and three patients with CD via Qiagen's RNeasy Plant Kit with DNase treatment according to manufacturer's protocol. RNA for total RNA sequencing was submitted to the Case Western Reserve Genomics Core for NEBNext Ultra II Directional RNA library prep and 75 bp paired‐end NextSeq. 550 High Output sequencing.

### 2.3 Bioinformatics

Bioinformatic analysis was performed by the Cleveland Institute for Computational Biology. Sequencing reads generated from the Illumina platform were assessed for quality and trimmed for adapter sequences using TrimGalore! v0.4.2 (Babraham Bioinformatics), a wrapper script for FastQC and cutadapt. Reads that passed quality control were then aligned to the human reference genome (GRCh37) using the STAR aligner v2.5.1. The alignment for the sequences were guided using the GENCODE annotation for hg19. The aligned reads were analyzed for differential expression using Cufflinks v2.2.1, a RNASeq analysis package which reports the fragments per kilobase of exon per million fragments mapped (FPKM) for each gene. Differential analysis report was generated using Cuffdiff. Differential genes were identified using a significance cutoff of *q* < 0.05. Differential gene expression was analyzed from six group comparisons: CD SubQ versus CD Mes, Healthy SubQ versus Healthy Mes, Healthy SubQ versus CD SubQ, Healthy Mes versus CD Mes, Healthy SubQ versus CD Mes, Healthy Mes versus CD SubQ (Magoč & Salzberg, 2011; Trapnell et al., 2010).

### 2.4 Pathway analysis

Pathway analysis of differentially expressed genes was performed with Qiagen's Ingenuity Pathway Analysis (IPA) software. All genes related to macrophages as identified by IPA's “Diseases and Functions” were sorted out of the main data set and a new Core gene expression analysis was run for all six groups. Focus was further placed on all the differentially expressed genes in “Activation of Macrophages” and “Migration of Macrophages” Functions. These genes were plotted as a heat map of the normalized FPKM *z*‐score for all groups. A table of the gene's log fold change and predicted effect on the function is listed in Supporting Information: Tables 1 and 2. The predicted effect on the functions were calculated in IPA using a *z*‐score statistical method. |z| > 2 were considered significant.

### 2.5 MSC isolation and culture

To isolate MSCs, fresh tissues were minced and digested with 1 mg/ml Collagenase from Clostridium histolyticum (Sigma‐Aldrich) at 37°C for 30 min. The digest was passed through a 100 µm cell strainer and centrifuged for 5 min at 1200 rpm. The cell pellets were resuspended in red blood cell lysis buffer and incubated for 1–2 min at 37°C. Cells were centrifuged again, resuspended in MSC media consisting of DMEM:F12 plus 10% fetal bovine serum (FBS), 1% glutamine (200 mM), and 1% penicillin/streptomycin (Cleveland Clinic Media Production Core) and cultured at 37°C with 5% CO_2_. Isolated MSCs from Mes and SubQ were stained with Vimentin (Abcam, ab92547), F‐actin (Abcam ab227216), and Ki‐67 (Cell Signaling, 9449). Bone marrow‐derived MSCs generated at the Hematopoietic Biorepository and Cellular Therapy Shared Resource at the Case Comprehensive Cancer Center were used as a control for MSC characterization.

THP‐1 cells were cultured in RPMI plus 10% FBS and 1% penicillin/streptomycin (Cleveland Clinic Media Production Core). Macrophage differentiation was achieved by adding 20 ng/ml phorbol‐12‐myristate‐13‐acetate (PMA) (EMD Millipore) for 24 h. Cells were cocultured with MSCs or EVs for indicated time points before harvest. Media were collected and centrifuged at 12,000 rpm for 5 min. Cells were scrapped in phosphate‐buffered saline (PBS) and pelleted at 5000 rpm for 5 min.

### 2.6 Extracellular vesicle isolation

MSC cultured media were collected from confluent cells (between passages 1–3) after 48–72 h. Media were centrifuged at 3000 rmp for 5 min to remove large cell debris and EVs were extracted and concentrated. Briefly, the cultured media were filtered through a 0.22 μm filter to remove cell debris and large vesicles, followed by ultracentrifugation at 30,000*g* for 20 min to pellet larger microvesicles. The supernatants were then subjected to ultracentrifugation at 120,000*g* for 3 h to sediment the MSC‐derived EVs. Supernatant was discarded and the EV pellet was resuspended in 200 µl 0.1 µM filtered PBS. For the TEM sample, 50 µl of EV suspension was mixed 1:1 with 4% 0.1 µM filtered PFA in PBS and stored at 4°C until processing. Remaining 50 µl aliquots were stored at −80°C. Transmission electron microscopy (TEM) sample processing and imaging was performed by the Cleveland Clinic Imaging Core on 2% PFA‐fixed EVs. Zetaview analysis was performed by the Cleveland Clinic Flow Cytometry Core.

### 2.7 Western blot

Patient tissues were sonicated in 300 µl RIPA lysis buffer plus Protease/Phosphatase inhibitor cocktails (Santa Cruz Biotechnologies). Tissue culture cell pellets were lysed by resuspension in lysis buffer. Samples were incubated on ice for 30 min and centrifuged at 12,000 rpm for 20 min at 4°C. Protein concentrations were determined with Pierce BCA Protein Assay Kit (Thermo Scientific) according to manufacturer's protocol. Ten to twenty micrograms of protein lysate was mixed with 2× Laemmli buffer (Bio‐rad Laboratories) and boiled at 95°C for 8 min. Proteins were separated according to their molecular mass on 4%–20% sodium dodecyl sulfate polyacrylamide gel electrophoresis gel (Genscript Biotech) and transferred to a nitrocellulose membrane (Bio‐Rad) via a TransBlot Turbo (Bio‐Rad). Primary antibodies for Phospho‐p44/42 MAPK (pERK) (4377), p44/42 MAPK (ERK) (9102), Phospho‐MEK1/2 (9154), MEK1/2 (8727), STAT3 (4904), cleaved caspase‐3 (9661), NOS (pan) (2977) were purchased from Cell Signaling Technologies and Direct horseradish peroxidase (HRP) anti‐β‐actin (664803) and Arginase 1 (678801) were purchased from Biolegend. Densitometry was performed and results were normalized to β‐actin. Normalized phosphorylated protein values were further normalized to total protein values.

### 2.8 Enzyme‐linked immunosorbent assay (ELISA)

ELISA plates were coated with 2 µg/ml capture (purified) antibodies overnight at 4°C. Wells were washed three times with TBST and blocked for 1 h with 1% bovine serum albumin. Block was removed and recombinant protein standards and samples were incubated for 2 h. Wells were washed three times and 2 µg/ml of biotynilated antibody was incubated for 1 h. Wells were washed three times and a 1:1000 dilution of HRP‐Avidin antibody was incubated for 40 min. Wells were washed three times and 75 µl TMB was added. The reaction was stopped with 75 µl 10% sulfuric acid and plates read at 450OD. Cytokine concentrations were determined from recombinant protein standard curve. Interleukin‐17A (IL‐17A) (576002), interferon‐γ IFN‐γ (575304), IL‐12 (572109), tumor necrosis factor α (TNF‐α) (570109), and IL‐6 (570809), anti‐IL‐17A (506901), anti‐IFN‐γ (505701), anti‐IL‐12 (501801), anti‐TNF‐α (502801), anti‐IL‐6 (79026)biotinylated anti‐IL‐17A (507001), biotinylated anti‐IFN‐γ (505803), biotinylated anti‐IL‐12 (508801), biotinylated anti‐TNF‐α (502903), and biotinylated anti‐IL‐6 (79027); and HRP‐Aviden (405103) antibodies were purchased from Biolegend.

### 2.9 Flow cytometry

THP‐1 derived macrophages were differentiated into M1 macrophages with 20 ng/ml IFN‐γ for 24 h. Cells were harvested with 0.5% Trypsin + 0.5 mM ethylenediaminetetraacetic acid for 20 min at 37°C. Cells were spun down at 6000 rpm for 3 min and washed twice with PBS. Cells were blocked with 2 µg/ml anti‐human CD16/32 FC‐blocking antibody (Biolegend) and stained for M1 macrophage surface marker with 2 µg/ml FITC‐anti‐human CD68 and anti‐human CD11b (Biolegend). The results were processed using BD FACSDIVA software (BD Bioscience) and data were extracted by FlowJo (BD Bioscience).

### 2.10 Statistical analysis

Statistical analyses were carried out using GraphPad Prism v.7 or JMP v.12 (SAS Institute). The data were checked to confirm normality and that groups had equal variance. One‐way analysis of variance (ANOVA) with Tukey's multiple comparison tests was employed to determine significant differences between sample groups. Results from these tests were reported as significant if *p* < 0.05, with results from these tests shown as mean ± SEM.

## 3 RESULTS

### 3.1 Active human CD is characterized by increased activation and migration of macrophages

**Figure 1:**
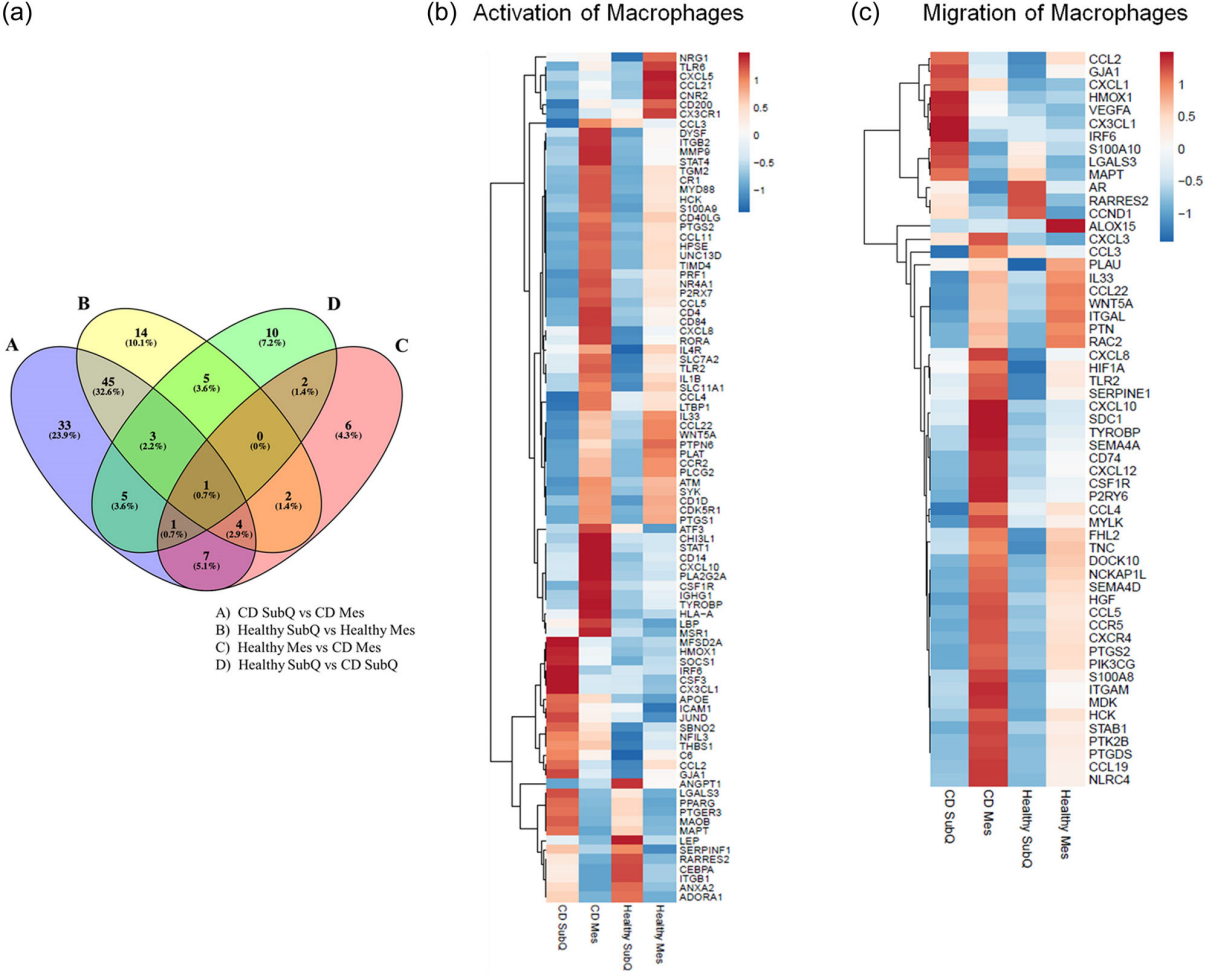
Adipose tissues' macrophage gene expression. (A) Venn diagram of significant differentially expressed genes related to macrophages. (B) Normalized FPKM *z*‐score heat map of significant differentially expressed genes related to the activation of macrophages in CD SubQ, CD Mes, Healthy SubQ, and Healthy Mes samples. (C) Normalized FPKM *z*‐score heat map of significant differentially expressed genes related to the migration of macrophages in CD SubQ, CD Mes, Healthy SubQ, and Healthy Mes samples. CD, Crohn's disease; FPKM, fragments per kilobase of exon per million fragments mapped.

**Figure 2:**
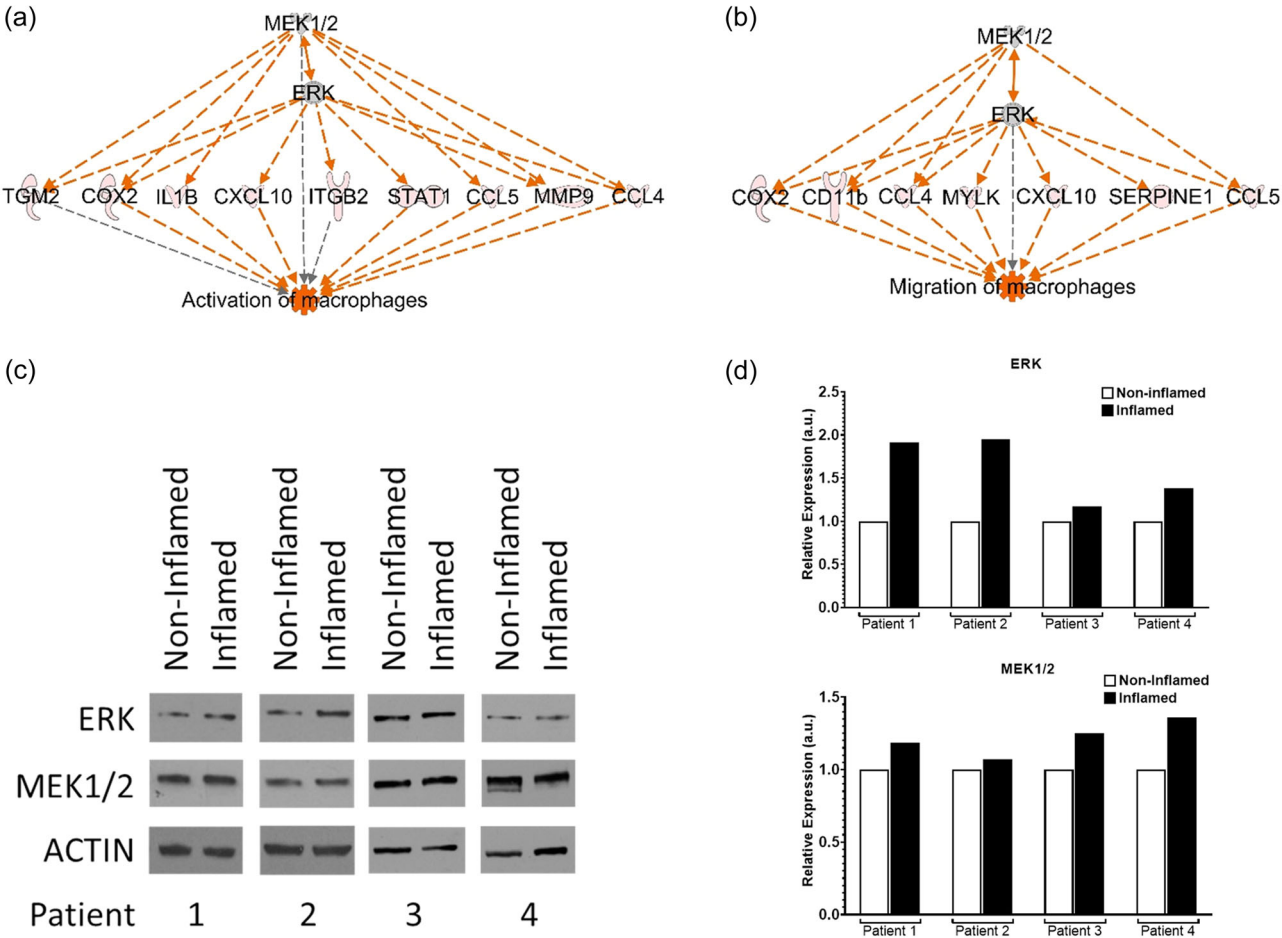
Upregulation of MEK/ERK signaling pathway activates inflamed Crohn's mesentery macrophage activation and migration. (A) Upstream regulation of differentially expressed genes by MEK/ERK on the activation of macrophages in CD SubQ versus CD Mes comparison. Grey, expressed; Red, upregulated; Orange, activated. (B) Upstream regulation of differentially expressed genes by MEK/ERK on the migration of macrophages in CD SubQ versus CD Mes comparison. Grey, expressed; Red, upregulated; Orange, activated. (C) Western blot analysis of CD patients' noninflamed and inflamed mesentery for ERK, MEK, and Actin. (D) Densitometry of ERK and MEK normalized to actin and the matching noninflamed tissue. CD, Crohn's disease.

The gene expression profiles of Healthy SubQ and Mes tissue and CD patients' noninflamed SubQ tissue and inflamed Mes tissue were examined for differential gene expression based upon six pairwise comparisons: CD SubQ versus CD Mes, Healthy SubQ versus Healthy Mes, Healthy SubQ versus CD SubQ, Healthy Mes versus CD Mes, Healthy SubQ versus CD Mes, and Healthy Mes versus CD SubQ. A total of 145 unique and shared significant differentially expressed genes related to macrophages were identified (Figure 1a). Consistent with the hypothesis that CD has an increased inflammatory state, pathway analysis demonstrated significant enrichment of these genes related to activation of macrophages and migration of macrophages functions. The expression profiles of the genes in these two functions were reversed in SubQ versus Mes tissue and demonstrated changes in the gene expression profile between Healthy Mes and CD Mes (Figure 1b,c). Supporting Information: Tables 1 and 2 show the significant fold change of each of the genes and their predicted effect on the activation or migration of macrophages. Significant *z*‐scores were associated with increased macrophage activation in CD SubQ versus CD Mes (*z*‐score 2.24, *p* < 0.001), Healthy SubQ versus Healthy Mes (*z*‐score 2.05, *p* < 0.001), Healthy Mes versus CD Mes (*z*‐score 2.37, *p* < 0.001), Healthy SubQ versus CD Mes (*z*‐score 3.09, *p* < 0.001) and increased macrophage migration in CD SubQ versus CD Mes (*z*‐score 3.04, *p* < 0.001), Healthy SubQ versus Healthy Mes (*z*‐score 3.16, *p* < 0.001), Healthy SubQ versus CD SubQ (*z*‐score 2.0, *p* < 0.001), Healthy SubQ versus CD Mes (*z*‐score 3.64, *p* < 0.001). While genes overlap in these groups, the fold changes in the inflamed states were exaggerated; 30 of the 62 differentially expressed genes in CD SubQ versus CD Mes as compared to only 24 of 44 genes in Healthy SubQ versus Healthy Mes have differential gene expression consistent with an increase in the activation of macrophages and for the majority of the genes that are shared within these two groups, the fold change is more excessive. Similarly, 29 of the 43 differentially expressed genes in CD SubQ versus CD Mes compared to 23 of 32 genes in Healthy SubQ versus Healthy Mes are consistent with an increase in the migration of macrophages with the majority of shared genes intensified in the inflamed state. Activation of the MEK/ERK signaling pathway was identified as a potential upstream regulator for several genes activating these two functions (Figure 2a). To interrogate this possibility, western blot analysis on matched noninflamed and inflamed mesentery from four CD patients was performed (Figure 2b,c). Under inflammatory conditions, the total protein content of MEK1/2 and ERK was increased.

### 3.2 MSC‐derived extracellular vesicles facilitate a monocyte to macrophage transition

**Figure 3:**
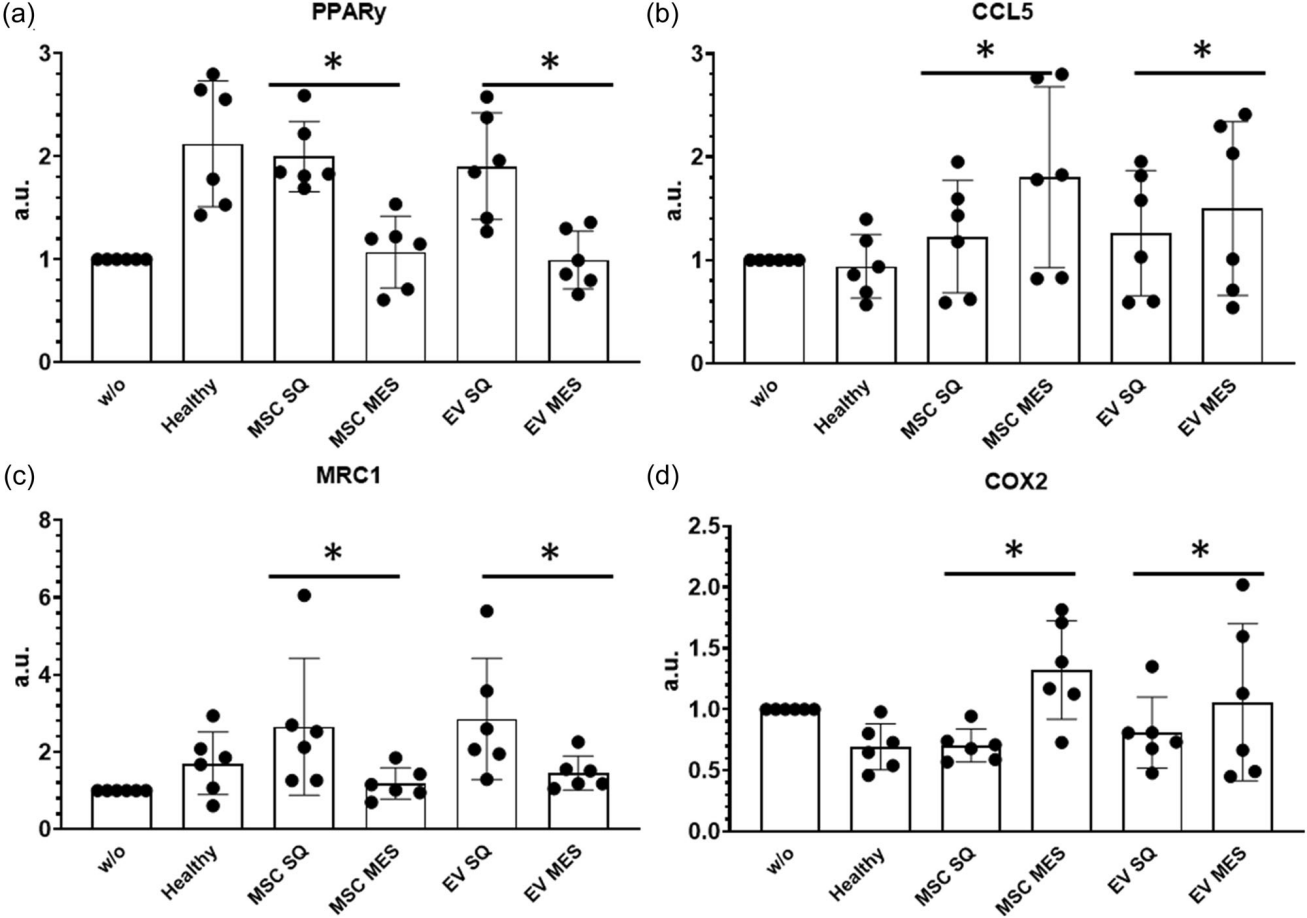
Inflamed Crohn's mesentery MSCs/extracellular vesicles regulated THP‐1 polarization to have higher M1 expression. RT‐qPCR analysis of M1/M2 gene expression in THP‐1 cells following 8 h stimulation with MSCs/EVs. M1 markers CCL5 (b) and COX2 (d) gene expression. M2 markers MRC1 (c) and PPARy (a) gene expression. *p < 0.05. ME, mesentery​​​​​; MSC, mesenchymal stem cells; RT‐qPCR, quantitative reverse‐transcription polymerase chain reaction; SQ, subcutaneous.

Macrophages have the capacity to polarize into proinflammatory (M1) and anti‐inflammatory macrophage (M2) populations. A total of 140 differentially expressed genes related to M1/M2 macrophages were identified. Again, the expression profiles were reversed in SubQ versus Mes tissue and demonstrated differences in expression between Healthy Mes and CD Mes (Supporting Information: Figure 1). Because there were no differences in specific function from this data, in vitro assays were used to further differentiate the phenotype. Monocytes are recruited to damaged and infected tissue when environmental stresses are sensed and differentiate into macrophages. THP‐1, a human monocyte line, responds to PMA stimulation and differentiates adherent macrophages. MSCs were isolated from noninflamed subcutaneous and inflamed mesenteric tissues to test the effect of MSCs and their EVs on macrophage differentiation. Isolated MSCs and EVs displayed similar characterization to normal bone‐marrow‐derived MSCs (Supporting Information: Figure 2). Inflamed MSCs and their produced EVs regulate PMA activated THP‐1 cells differentiation into macrophages as depicted by the concomitant increased M1 associated transcript levels (CCL5, COX2) and reduced M2 associated transcript levels (MRC1, PPARƳ) (Figure 3). In addition, THP‐1 monocytes are more phagocytic as compared to macrophages, which evolve to perform more specialized cytokine production function. An increase in M1 and decrease in M2 phagocytic index was noted in PMA differentiated inflamed MSCs/EVs treated macrophages as compared to THP‐1 monocytes as reported. An overall analysis of undifferentiated THP‐1‐MSC/EV cocultures indicates a possible transition of naïve THP‐1 monocytes to the macrophage‐like state upon MSCs/EVs exposure.

### 3.3 Inflamed MSC derived extracellular vesicles induce high protein expression of M1 polarization

**Figure 4:**
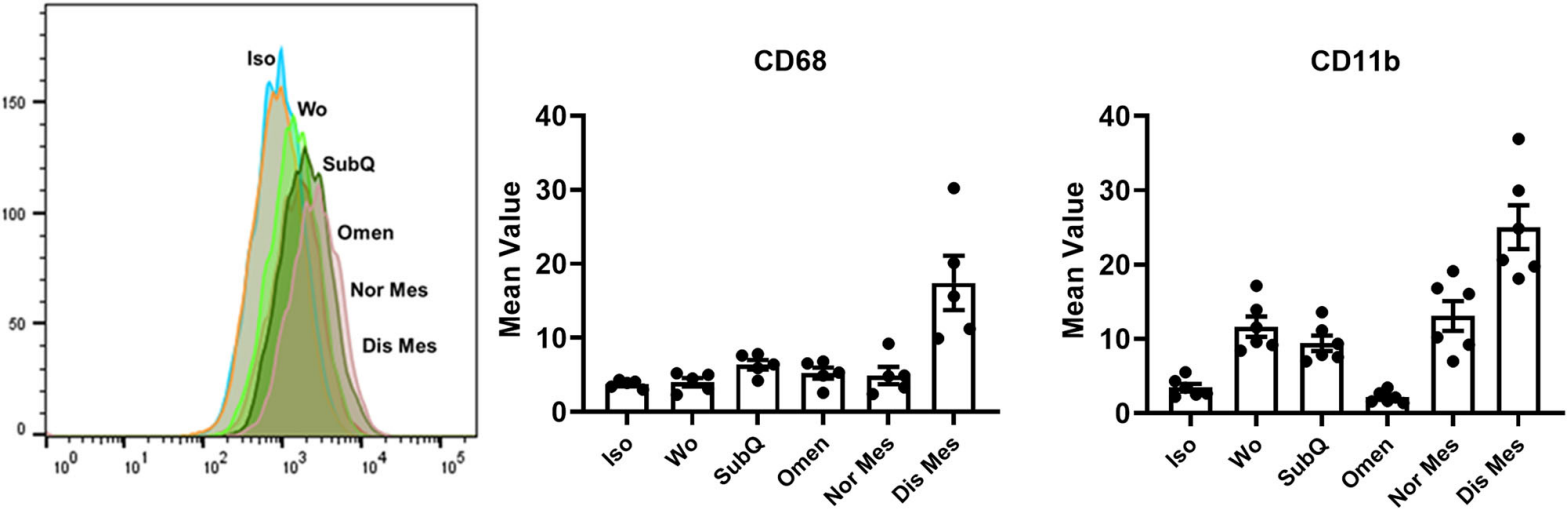
Immunophenotyping macrophages. Flow cytometry analysis of undifferentiated and differentiated THP‐1 cells treated by extracellular vesicles, CD11b expression and CD68 expression. Flow cytometry data and micrographs represent results from three individual bat derived tissues. Data are presented as mean ± SEM from three individual bat derived EVs. D/Dis, diseased tissue; N/Norm, normal tissue; O/Omen, omentem; S/SubQ, subcutaneous.

The balance of tolerogenic or differentiation function from proinflammatory microenvironments is characterized by the balance of macrophage stability, especially given that these populations have proven to be in either M1 or M2 cohorts (Belkaid & Hand, 2014). To analyze whether MSC derived EVs can modify the monocyte state, EVs were cocultured with the human monocyte line THP‐1 and evaluated for the induction of CD68 and CD11b cell surface expression. Cell surface expression was noted in macrophages and EV‐THP‐1 cocultures (Figure 4). THP‐1 cells with diseased Mes EVs treatment displayed a significant accumulation of CD68+ and CD11b+ cells as compared to both normal Mes EVs and SubQ EVs. Notably, whereas CD68− and CD11b− cells in normal Mes EVs or SubQ EVs treatment groups expressed high levels of Arg‐1, CD68+ cells were found in diseased Mes EVs and had an Arg‐1^−/low^ profile, consistent with a macrophage balance state.

### 3.4 Inflamed extracellular vesicles suppress the levels of proinflammatory cytokines and promote the anti‐inflammatory cytokine secretion

**Figure 5:**
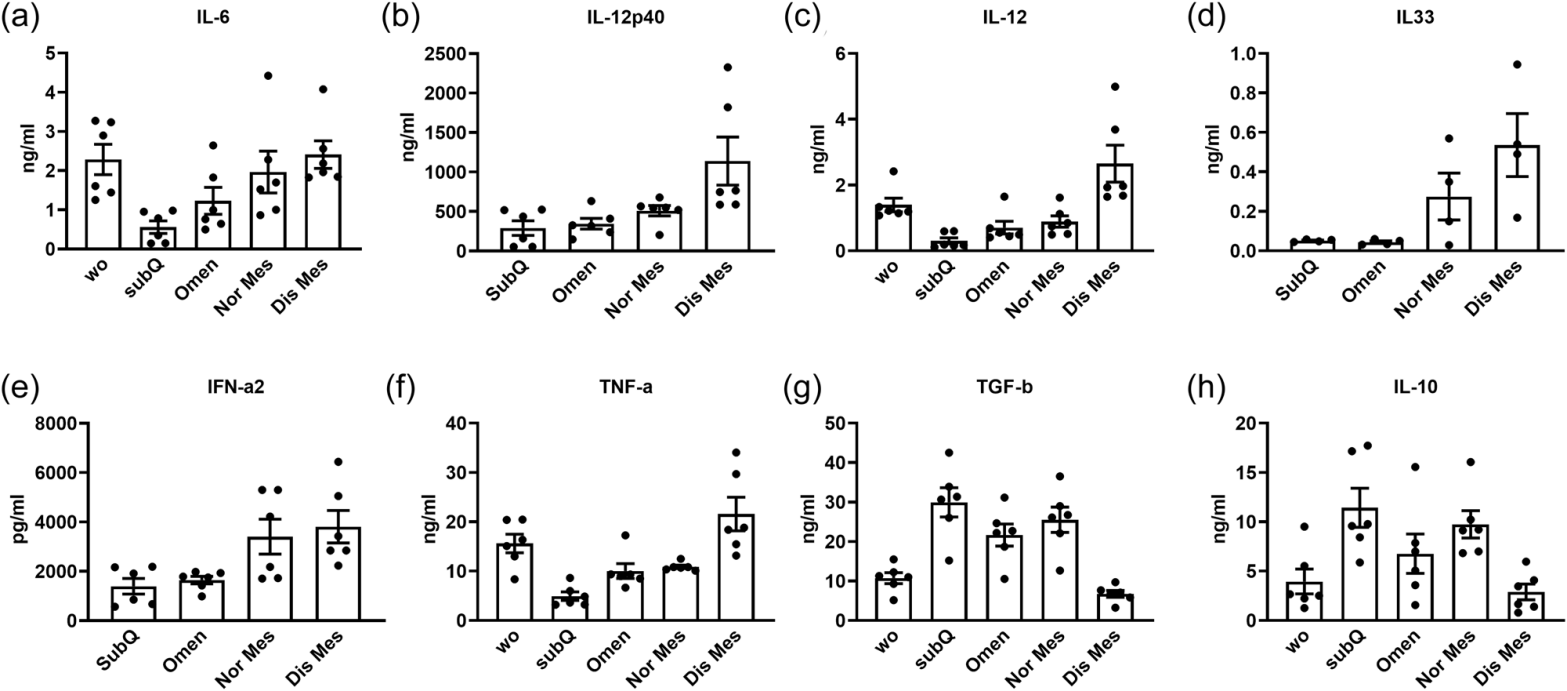
Extracellular vesicles favor macrophage reprogramming during the resolution of inflammation. THP‐1 monocytes were differentiated under the PMA conditions with extracellular vesicles treatment over 72 h. Culture supernatants were then collected and proinflammatory cytokine secretion were determined by selective ELISA. Levels of IL‐6 (a), IL‐12p40 (b), IL‐12 (c), IL33 (d), IFN‐a2 (e) are significantly increased in the diseased mesentery extracellular vesicles group compared to the other groups while TNF‐a (f), TGF‐b (g), and IL‐10 (h) are significantly decreased in the diseased mesentery group. Results are mean ± SEM from four independent experiments. *p< 0.05. ELISA, enzyme‐linked immunosorbent assay; IL‐6, interleukin 6; PMA, phorbol‐12‐myristate‐13‐acetate; TNF‐a, tumor necrosis factor‐a.

The M1 macrophage associated cytokines, IL‐6, IL‐12, and TNF polymorphisms are associated with a CD predisposition, indicating that proinflammatory cytokine development is linked to the susceptibility of various illnesses (Lamb et al., 2019; Panaccione et al., 2019). ELISA was used to test the key M1/M2 cytokines secretions (Figure 5). M1 macrophages' cytokines including TNF‐a, IL‐6, IL‐12, IL‐12p40, IL‐33, and IFN‐a2 were significantly increased after diseased Mes EVs treatment, while the key cytokines of M2 macrophage, TGF‐b and IL‐10 were reduced, confirming that these cytokines are a hallmark of disease activity and EVs have the predicted capacity to regulate macrophage differentiation.

### 3.5 Phosphorylation of kinase and intracellular signaling pathways in the regulation of macrophage differentiation

**Figure 6:**
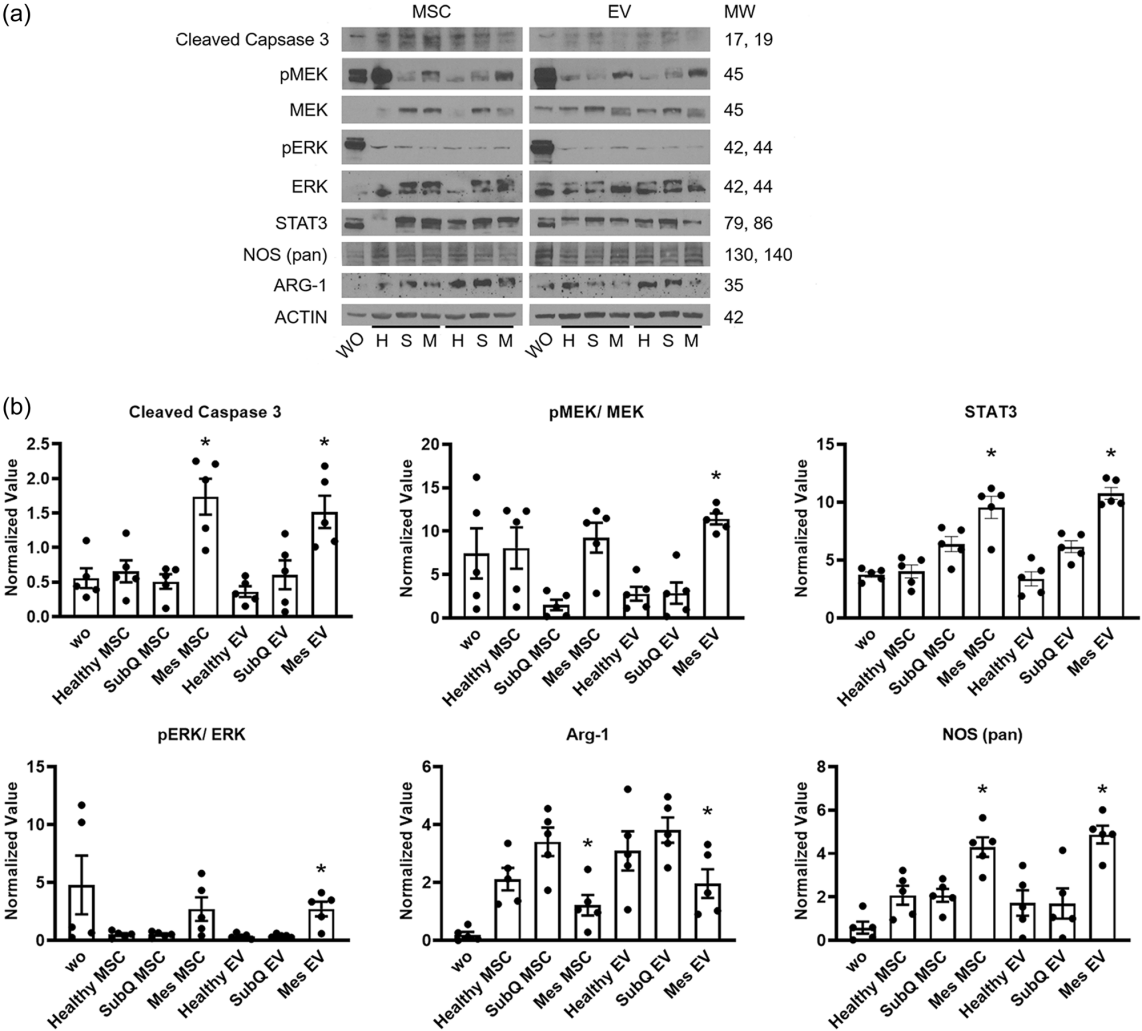
Enhanced cleaved caspase 3, MEK, ERK, STAT3, and suppressed Arg‐1 signaling in inflamed extracellular vesicles incubated macrophages. THP‐1 cells were exposed to inflamed, subcutaneous or noninflamed MSCs or extracellular vesicles for 72 h in macrophage differentiation conditions before protein harvest and western blot analysis. (a) The levels of cleaved caspase 3, pMEK, MEK, pERK, ERK, STAT3, and ARG‐1 after MSCs treatment were determined by immunoblotting. (b) The levels of cleaved caspase 3, pMEK, MEK, pERK, ERK, STAT3, and ARG‐1 after extracellular vesicles treatment were determined by immunoblotting. Relative band intensities were quantified from five individual experiments and measured by densitometry analysis. Beta‐actin was used as a loading control. Phosphorylated proteins were further normalized by total protein values. Mes, diseased mesentery; MSC, mesenchymal stem cells; Subq, subcutaneous; WO, without treatment. *n* = 3 patients. *p < 0.05.

In vitro M1/M2 stimulation is transduced through the JAK‐STAT and ERK pathways (Bao et al., 2013; Busch‐Dienstfertig & Gonzalez‐Rodriguez, 2013; Pesu et al., 2000; Sasi et al., 2014; Vainchenker & Constantinescu, 2013). Furthermore, our preceding research indicated that the MSC/EV on DSS‐induced murine colitis model may involve the JAK/Caspase signaling pathway. Therefore, the phosphorylation of kinases in the signaling pathways were evaluated to obtain clear evidence of activation. After incubation with MSC/EVs for 72 h, levels of cleaved caspase 3, phosphorylated ERK1/2, phosphorylated MEK, and STAT3 were all increased in the diseased Crohn's mesentery suggesting M1 polarization activated these signaling pathways (Figure 6).

## 4 DISCUSSION

CD exhibits significant production of proinflammatory cytokines from the local recruitment of proinflammatory cells which can result in local tissue damage (Rovedatti et al., 2009). Cytokine genetic polymorphisms and the balance between M1/M2 macrophages have been associated with many inflammatory diseases, suggesting variation in the ability to regulate proinflammatory cells and their subsequential cytokine production may result in disease susceptibility (Balding et al., 2004; Cantor et al., 2005). Pathognomonic to CD is inflamed and hypervascularized mesentery which wraps around the bowel wall, aka creeping fat, and has recently been seen as an active player in the pathophysiology of CD. However, the reason why diseased mesentery plays an active role in disease progression remains unknown (Coffey et al., 2018; Ha et al., 2020). We herein found that both MSCs and their associated EVs derived from diseased mesentery characteristic of CD may contribute to an ongoing inflammation and disease progression due to a M1 proinflammatory phenotype.

Total RNA sequencing was used to assess the mRNA expression signature of macrophage related genes in adipose tissue from healthy patients, adipose tissue from CD subcutaneous layer, and adipose tissue from diseased Crohn's mesentery. Pathway analysis indicated an increase in the activation and migration of macrophages with distinct expression profiles in between healthy versus Crohn's subcutaneous tissue versus CD mesentery tissue. Results from other papers and our own previous investigation have reported that the elevated activity of the mitogen‐activated protein kinase (MAPK) signaling cascade is found in the majority of mouse colon tissue and is known to regulate proliferation, survival and invasion of macrophage populations (Guereño et al., 2020; Hernandez‐Padilla et al., 2020; Hernandez‐Silva et al., 2020; Yi et al., 2020; Zhao et al., 2020). This process involves inflammatory factors, such as MEK and ERK. Consistent with these findings, we found that MEK and ERK act as potential upstream regulators of several differentially expressed genes related to the activation and migration of macrophages and were able to confirm elevated protein levels of MEK and ERK in inflamed compared to noninflamed mesentery of CD patients.

While MSCs are known to be a safe and effective therapeutic for the treatment of perianal CD (Cho et al., 2015; de la Portilla et al., 2013; Garcia‐Olmo et al., 2009; Pan et al., 2014; Panés et al., 2016), likely due to their anti‐inflammatory and immunomodulatory properties which promote tissue repair (Kinchen et al., 2018; Mao et al., 2017; Soontararak et al., 2018; Turse et al., 2018; Zhang et al., 2017), the source of MSCs is critical to the phenotype and function of the cell. When MSCs are extracted from diseased mesentery they appear to be proinflammatory, largely due to their effect on macrophage polarization. How this could be reversed remains unknown. Given there is an increasing interest in using extracellular vesicles from MSCs as a therapeutic in place of MSCs (Phinney & Pittenger, 2017; Rackov et al., 2018; Ren et al., 2019; Spees et al., 2016), we also investigated the if MSCs and EVs were consistent in their ability to stimulate macrophage polarization. Interestingly, we found MSCs and their associated extracellular vesicles were equivalent and consistent in macrophage polarization, underscoring the potential utility of extracellular vesicles as a therapeutic. Both the MSC and MSC‐derived extracellular vesicles from Crohn's mesentery resulted in M1 macrophage polarization, whereas MSCs and MSC‐derived extracellular vesicles from healthy patients resulted in M2 macrophage polarization. Thus, MSC‐derived extracellular vesicles could be a potential future therapeutic for the treatment of CD, whereas the MSCs in creeping fat may continue to propagate and worsen the inflammatory burden in CD.

There are limitations to our study worth mentioning. First, we did not look to see if healthy MSCs and healthy MSC‐derived extracellular vesicles reversed macrophage polarization in the setting of inflamed states within an in vivo model. Second, we did not investigate whether the concentration of MSCs or extracellular vesicles affected macrophage polarization to understand if this finding was dose‐dependent and thus suggesting a critical level of inflammation would be required to see the same results. Third, we did not investigate the systemic effects of the MSC and MSC‐derived extracellular vesicles which would be important in future investigations.

In conclusion, diseased Crohn's mesentery exhibits a proinflammatory state through the polarization of macrophages to a M1 phenotype. This may be driven by MSCs in the resident tissue and their associated extracellular vesicles resulting in a proinflammatory phenotype. Conversely, healthy donor‐derived MSCs and their associated extracellular vesicles have the ability to drive monocytes to a M2 subset, effectively reversing an inflammatory phenotype. This mechanism supports why MSCs may be an effective therapeutic in CD and highlights extracellular vesicles as a novel therapeutic for further exploration.

## AUTHOR CONTRIBUTIONS

We confirm that the manuscript has been read and approved by all named authors and that there are no other persons who satisfied the criteria for authorship but are not listed. We further confirm that the order of authors listed in the manuscript has been approved by all of us. Neda Dadgar, Yan Li, and Jessica Altemus carried out basic science experiments in the study. Yan Li and Neda Dadgar wrote the draft of the manuscript with support from Amy L. Lightner, Amy L. Lightner, Neda Dadgar, and Jessica Altemus performed the analysis. Amy L. Lightner supervised the project and wrote final draft of manuscript. All authors approved final manuscript.

## CONFLICTS OF INTEREST

Amy Lightner consults for Ossium, Takeda, Mesoblast.

## Supporting information

Supporting information.

Supporting information.

Supporting information.
