# Supplementary figures and images for: Adipose tissue‐derived mesenchymal stem cells' acellular product extracellular vesicles as a potential therapy for Crohn's disease
Source: J Cell Physiol. 2022 May 6;237(7):3001–11. doi: 10.1002/jcp.30756 (PMC9544647; doi:10.1002/jcp.30756)

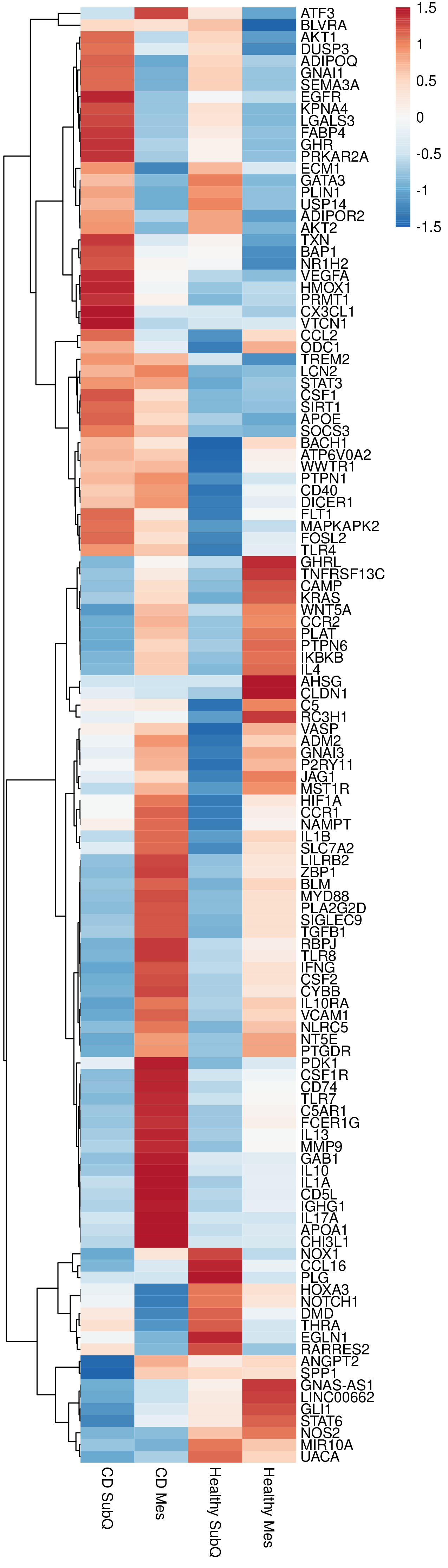

Supplement: Supplementary file 1 — Supporting information. [file JCP-237-3001-s002.tiff]

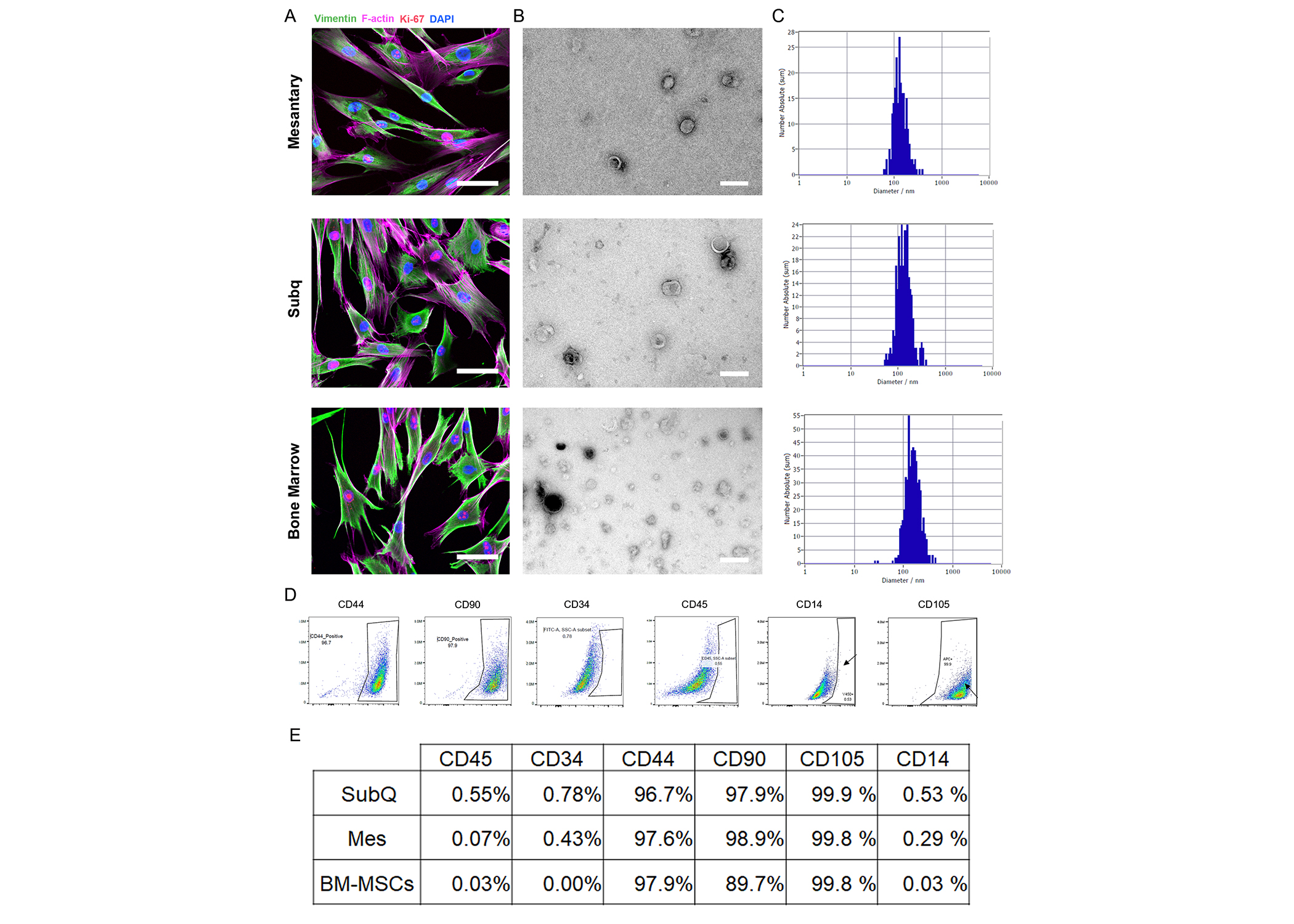

Supplement: Supplementary file 2 — Supporting information. [file JCP-237-3001-s001.tiff]
