# Supplementary material for: Adipose tissue‐derived mesenchymal stem cells' acellular product extracellular vesicles as a potential therapy for Crohn's disease
Source: J Cell Physiol. 2022 May 6;237(7):3001–11. doi: 10.1002/jcp.30756 (PMC9544647; doi:10.1002/jcp.30756)
Supplement: Supplementary file 3 — Supporting information. [file JCP-237-3001-s003.docx]

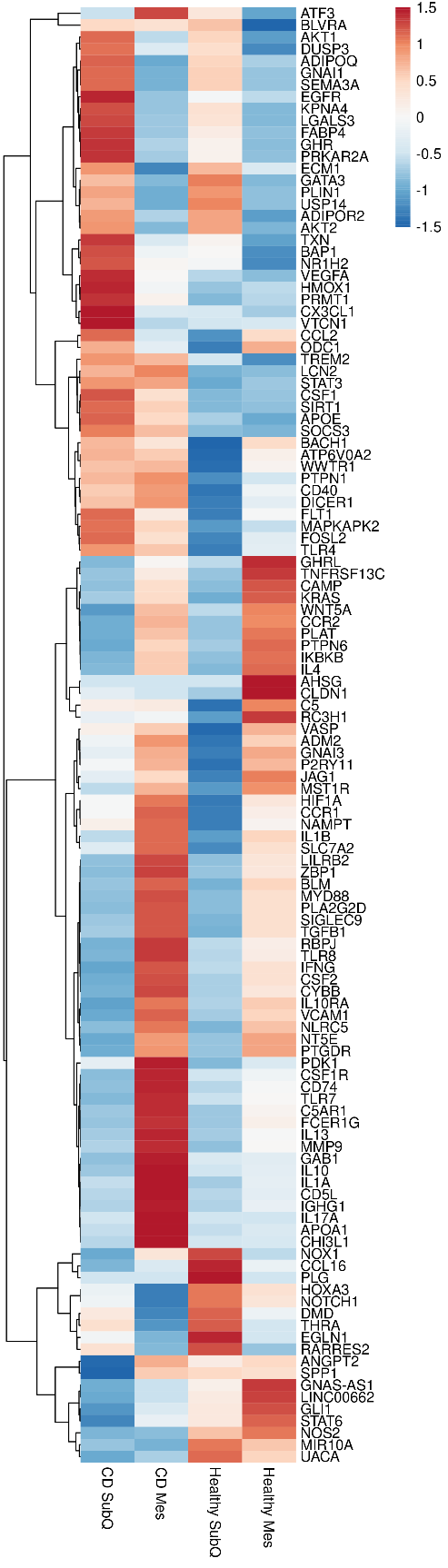


Figure S1, Normalized FPKM z-score heat map of significant differentially expressed genes related to M1 and M2 Macrophages in CD SubQ, CD Mes, Healthy SubQ, and Healthy Mes samples.

Table S1- Migration of Macrophages

|  | CD SubQ vs CD Mes | | Healthy SubQ vs Healthy Mes | | Healthy SubQ vs CD SubQ | | Healthy Mes vs CD Mes | | Healthy SubQ vs CD Mes | | Healthy Mes vs CD SubQ | |
| --- | --- | --- | --- | --- | --- | --- | --- | --- | --- | --- | --- | --- |
| Genes | FC | P | FC | P | FC | P | FC | P | FC | P | FC | P |
| ALOX15 |  |  | 5.308 | ↑ |  |  | -3.886 | ↓ |  |  | -6.901 | ↓ |
| AR | -1.289 | ↓ | -0.942 | ↓ |  |  | -0.899 | ↓ | -1.841 | ↓ |  |  |
| CCL19 | 2.995 | ↔ | 2.16 | ↔ |  |  |  |  | 3.112 | ↔ | -2.043 | ↔ |
| CCL22 | 2.597 | ↑ | 1.669 | ↑ |  |  |  |  |  |  | -2.84 | ↓ |
| CCL2 |  |  |  |  | 1.196 | ↑ |  |  |  |  |  |  |
| CCL3 | 2.274 | ↑ |  |  | -2.017 | ↓ |  |  |  |  |  |  |
| CCL4 | 2.469 | ↑ |  |  | -1.611 | ↓ |  |  |  |  | -2.092 | ↓ |
| CCL5 | 3.326 | ↑ | 1.957 | ↑ |  |  |  |  | 2.665 | ↑ | -2.617 | ↓ |
| CCND1 |  |  | -1.795 | ↓ |  |  |  |  | -1.304 | ↓ | 1.404 | ↑ |
| CCR5 | 3.199 | ↑ |  |  |  |  |  |  | 2.301 | ↑ | -2.585 | ↓ |
| CD74 | 1.395 | ↔ |  |  |  |  |  |  | 1.202 | ↔ |  |  |
| CSF1R | 1.348 | ↑ |  |  |  |  |  |  | 1.062 | ↑ |  |  |
| CX3CL1 | -1.065 | ↓ |  |  | 1.091 | ↑ |  |  |  |  | 1.382 | ↑ |
| CXCL1 |  |  |  |  | 2.917 | ↔ | 1.797 | ↔ | 2.42 | ↔ | 2.295 | ↔ |
| CXCL10 | 2.22 | ↑ |  |  |  |  | 2.357 | ↑ | 3.356 | ↑ |  |  |
| CXCL12 | 1.463 | ↑ |  |  |  |  |  |  | 1.282 | ↑ |  |  |
| CXCL3 |  |  |  |  |  |  | 2.653 | ↑ | 1.962 | ↑ | 2.097 | ↑ |
| CXCL8 |  |  | 2.237 | ↔ | 2.174 | ↔ |  |  | 3.253 | ↔ |  |  |
| CXCR4 | 4.035 | ↔ | 2.714 | ↔ |  |  |  |  | 3.35 | ↔ | -3.399 | ↔ |
| DOCK10 | 2.331 | ↑ | 2.053 | ↑ |  |  |  |  | 2.36 | ↑ | -2.024 | ↓ |
| FHL2 |  |  | 1.153 | ↓ |  |  |  |  | 1.349 | ↓ |  |  |
| GJA1 |  |  |  |  | 0.927 | ↑ |  |  |  |  |  |  |
| HCK | 1.213 | ↑ | 1.041 | ↑ |  |  |  |  | 1.451 | ↑ |  |  |
| HGF | 1.291 | ↑ |  |  |  |  |  |  |  |  |  |  |
| HIF1A |  |  | 1.055 | ↑ | 0.916 | ↑ |  |  | 1.375 | ↑ |  |  |
| HMOX1 | -1.309 | ↓ |  |  | 2.795 | ↑ | 0.967 | ↑ | 1.486 | ↑ | 2.276 | ↑ |
| IL33 | 1.201 | ↑ | 0.817 | ↑ |  |  |  |  |  |  | -1.307 | ↓ |
| IRF6 | -1.82 | ↓ |  |  | 1.46 | ↑ |  |  |  |  | 1.563 | ↑ |
| ITGAL | 3.199 | ↑ | 2.378 | ↑ |  |  |  |  | 2.052 | ↑ | -3.525 | ↓ |
| ITGAM | 0.856 | ↑ |  |  |  |  |  |  | 1.036 | ↑ |  |  |
| LGALS3 | -1.091 | ↓ | -0.797 | ↓ |  |  |  |  |  |  | 1.199 | ↑ |
| MAPT | -2.075 | ↓ |  |  |  |  |  |  | -1.748 | ↓ | 1.802 | ↑ |
| MDK |  |  |  |  |  |  |  |  | 1.232 | ↑ |  |  |
| MYLK | 1.773 | ↑ |  |  |  |  |  |  |  |  |  |  |
| NCKAP1L | 2.004 | ↑ | 1.433 | ↑ |  |  |  |  | 1.764 | ↑ | -1.673 | ↓ |
| NLRC4 | 2.095 | ↓ |  |  |  |  |  |  | 2.306 | ↓ |  |  |
| P2RY6 | 1.9 | ↔ |  |  |  |  |  |  |  |  |  |  |
| PIK3CG | 2.146 | ↑ | 1.273 | ↑ |  |  |  |  | 1.727 | ↑ | -1.693 | ↓ |
| PLAU |  |  | 1.351 | ↑ | 1.041 | ↑ |  |  | 1.208 | ↑ |  |  |
| PTGDS | 4.031 | ↑ | 3.223 | ↑ |  |  | 0.959 | ↑ | 4.182 | ↑ | -3.072 | ↓ |
| PTGS2 | 2.837 | ↑ | 1.716 | ↑ |  |  |  |  | 2.194 | ↑ | -2.36 | ↓ |
| PTK2B | 1.877 | ↑ | 1.303 | ↑ |  |  |  |  | 1.925 | ↑ | -1.255 | ↓ |
| PTN | 4.213 | ↑ | 4.156 | ↑ |  |  |  |  | 3.947 | ↑ | -4.421 | ↓ |
| RAC2 | 2.121 | ↑ | 2.335 | ↑ |  |  |  |  | 2.081 | ↑ | -2.375 | ↓ |
| RARRES2 | -0.833 | ↓ | -1.158 | ↓ |  |  |  |  | -1.229 | ↓ |  |  |
| S100A10 | -1.476 | ↓ |  |  |  |  |  |  | -0.952 | ↓ | 1.081 | ↑ |
| S100A8 | 1.329 | ↑ | 1.146 | ↑ |  |  |  |  | 1.764 | ↑ |  |  |
| SDC1 | 2.329 | ↑ | 3.515 | ↑ | 3.557 | ↑ | 2.37 | ↑ | 5.885 | ↑ |  |  |
| SEMA4A | 2.555 | ↑ | 1.422 | ↑ |  |  | 1.483 | ↑ | 2.905 | ↑ |  |  |
| SEMA4D | 2.675 | ↓ | 1.994 | ↓ |  |  |  |  | 2.459 | ↓ | -2.21 | ↑ |
| SERPINE1 | 1.034 | ↑ | 2.296 | ↑ | 1.955 | ↑ |  |  | 2.989 | ↑ |  |  |
| STAB1 | 1.211 | ↑ |  |  |  |  |  |  |  |  |  |  |
| TLR2 |  |  | 1.253 | ↑ |  |  |  |  | 1.687 | ↑ |  |  |
| TNC |  |  | 1.02 | ↑ |  |  |  |  | 1.135 | ↑ |  |  |
| TYROBP | 1.273 | ↑ |  |  |  |  | 0.959 | ↑ | 1.334 | ↑ |  |  |
| VEGFA |  |  |  |  | 1.332 | ↑ |  |  |  |  | 1.582 | ↑ |
| WNT5A | 2.773 | ↑ | 1.599 | ↑ | -1.402 | ↓ |  |  | 1.372 | ↑ | -3 | ↓ |

FC = Log Fold-change, P = Predicted affect, **↑** = Increased, ↓ = Decreased, **↔** = Affected

Table S2-Activation of Macrophages

|  | CD SubQ vs CD Mes | | Healthy SubQ vs Healthy Mes | | Healthy SubQ vs CD SubQ | | Healthy Mes vs CD Mes | | Healthy SubQ vs CD Mes | | Healthy Mes vs CD SubQ | |
| --- | --- | --- | --- | --- | --- | --- | --- | --- | --- | --- | --- | --- |
| Genes | FC | P | FC | P | FC | P | FC | P | FC | P | FC | P |
| ADORA1 |  |  | -1.592 | ↑ |  |  |  |  |  |  |  |  |
| APOE |  |  |  |  | 1.436 | ↑ | 1.579 | ↑ |  |  | 1.941 | ↑ |
| ANGPT1 |  |  |  |  | -1.19 | ↑ |  |  |  |  |  |  |
| ANXA2 | -1.064 | ↔ | -1.106 | ↔ |  |  |  |  |  |  |  |  |
| ATF3 | 0.941 | ↓ |  |  |  |  | 1.337 | ↓ |  |  |  |  |
| ATM | 1.826 | ↓ | 1.079 | ↓ |  |  |  |  | 1.202 | ↓ | -1.703 | ↑ |
| C6 |  |  |  |  | 1.394 | ↑ |  |  |  |  |  |  |
| CCL11 |  |  | ∞ | ↑ |  |  |  |  | ∞ | ↑ |  |  |
| CCL2 |  |  |  |  | 1.196 | ↑ |  |  |  |  |  |  |
| CCL21 | 4.381 | ↑ | 4.796 | ↑ |  |  | -1.417 | ↓ | 3.379 | ↑ | -5.798 | ↓ |
| CCL22 | 2.597 | ↑ | 1.669 | ↑ |  |  |  |  |  |  | -2.84 | ↓ |
| CCL3 | 2.274 | ↑ |  |  | -2.017 | ↓ |  |  |  |  |  |  |
| CCL4 | 2.469 | ↑ |  |  | -1.611 | ↓ |  |  |  |  | -2.092 | ↓ |
| CCL5 | 3.326 | ↑ | 1.957 | ↑ |  |  |  |  | 2.665 | ↑ | -2.617 | ↓ |
| CCR2 | 2.984 | ↑ | 2.368 | ↑ |  |  |  |  | 2.203 | ↑ | -3.15 | ↓ |
| CD14 | 1.066 | ↑ |  |  |  |  | 0.983 | ↑ | 1.36 | ↑ |  |  |
| CD1D |  |  | 1.9 | ↑ |  |  |  |  | 1.938 | ↑ |  |  |
| CD200 |  |  |  |  |  |  |  |  |  |  | -1.244 | ↑ |
| CD4 | 1.649 | ↑ |  |  |  |  |  |  | 1.405 | ↑ | -0.974 | ↓ |
| CD40LG | 3.59 | ↔ | 3.258 | ↔ |  |  |  |  | 3.613 | ↔ | -3.235 | ↔ |
| CD84 | 2.104 | ↔ | 1.111 | ↔ |  |  |  |  | 1.867 | ↔ | -1.348 | ↔ |
| CDK5R1 | 2.086 | ↓ | 1.837 | ↓ |  |  |  |  | 1.942 | ↓ | -1.981 | ↑ |
| CEBPA | -1.269 | ↓ | -1.379 | ↓ |  |  |  |  |  |  | 0.804 | ↑ |
| CHI3L1 | 3.77 | ↑ |  |  |  |  | 2.634 | ↑ | 2.778 | ↑ |  |  |
| CLEC4E | 1.54 | ↑ |  |  |  |  |  |  |  |  |  |  |
| CNR2 |  |  | 6.298 | ↓ |  |  | -1.778 | ↑ | 4.52 | ↓ |  |  |
| CR1 | 2.659 | ↓ | 2.383 | ↓ |  |  |  |  | 2.987 | ↓ | -2.055 | ↑ |
| CSF1R | 1.348 | ↔ |  |  |  |  |  |  |  |  |  |  |
| CSF3 | -1.495 | ↓ |  |  |  |  |  |  |  |  | 2.229 | ↑ |
| CX3CL1 | -1.065 | ↓ |  |  | 1.091 | ↑ |  |  |  |  | 1.382 | ↑ |
| CX3CR1 |  |  |  |  |  |  |  |  |  |  | -2.417 | ↔ |
| CXCL10 | 2.22 | ↑ |  |  |  |  | 2.357 | ↑ | 3.356 | ↑ |  |  |
| CXCL5 |  |  |  |  |  |  |  |  |  |  | -4.137 | ↔ |
| CXCL8 |  |  | 2.237 | ↑ | 2.174 | ↑ |  |  | 3.253 | ↑ |  |  |
| GJA1 |  |  |  |  | 0.927 | ↓ |  |  |  |  |  |  |
| HCK | 1.213 | ↑ | 1.041 | ↑ |  |  |  |  | 1.451 | ↑ |  |  |
| HLA-A |  |  |  |  |  |  | 1.101 | ↔ | 1.32 | ↔ |  |  |
| HMOX1 | -1.309 | ↓ |  |  | 2.795 | ↑ | 0.967 | ↑ | 1.486 | ↑ | 2.276 | ↑ |
| HPSE | 2.017 | ↑ |  |  |  |  |  |  | 1.793 | ↑ | -1.646 | ↓ |
| ICAM1 |  |  |  |  |  |  | 1.412 | ↑ |  |  | 1.934 | ↑ |
| IGHG1 | 6.367 | ↑ | 5.111 | ↑ |  |  |  |  | 7.282 | ↑ | -4.195 | ↓ |
| IL10 | 2.868 | ↓ |  |  |  |  |  |  |  |  |  |  |
| IL1B | 1.519 | ↑ | 2.313 | ↑ |  |  |  |  | 2.677 | ↑ |  |  |
| IL33 | 1.201 | ↑ | 0.817 | ↑ |  |  |  |  |  |  | -1.307 | ↓ |
| IL4R |  |  | 1.168 | ↑ |  |  | 2.734 | ↑ | 1.269 | ↑ |  |  |
| IRF6 | -1.82 | ↓ |  |  | 1.46 | ↑ |  |  |  |  | 1.563 | ↑ |
| ITGB2 | 1.781 | ↔ | 1.077 | ↔ |  |  |  |  | 1.896 | ↔ | -0.961 | ↔ |
| JUND |  |  |  |  |  |  |  |  |  |  | 1.028 | ↑ |
| LBP |  |  |  |  |  |  |  |  | 1.753 | ↑ | 1.948 | ↑ |
| LEP | -1.225 | ↓ | -1.951 | ↓ | -1.423 | ↓ |  |  |  |  |  |  |
| LGALS3 | -1.091 | ↓ | -0.797 | ↓ |  |  |  |  |  |  | 1.199 | ↑ |
| LTBP1 | 1.265 | ↑ |  |  |  |  |  |  |  |  | -1.031 | ↓ |
| MAOB | -1.185 | ↓ | -0.832 | ↓ |  |  |  |  |  |  | 1.161 | ↑ |
| MAPT | -2.075 | ↔ |  |  |  |  |  |  |  |  | 1.802 | ↔ |
| MFSD2A | -2.154 | ↑ |  |  | 5.437 | ↓ |  |  | 3.282 | ↓ | 3.379 | ↓ |
| MMP9 | 1.991 | ↑ | 1.359 | ↑ |  |  |  |  | 2.373 | ↑ |  |  |
| MSR1 |  |  |  |  |  |  | 1.367 | ↑ |  |  |  |  |
| MYD88 | 0.934 | ↑ |  |  |  |  |  |  |  |  |  |  |
| NFIL3 |  |  |  |  | 1.119 | ↔ |  |  |  |  |  |  |
| NR4A1 | 1.58 | ↓ |  |  |  |  |  |  | 1.182 | ↓ |  |  |
| NRG1 |  |  | 2.833 | ↑ | 2.088 | ↑ |  |  | 2.196 | ↑ |  |  |
| P2RX7 | 1.963 | ↔ |  |  |  |  |  |  | 1.371 | ↔ | -1.517 | ↔ |
| PLA2G2A | 1.381 | ↔ |  |  |  |  | 1.388 | ↔ | 1.621 | ↔ |  |  |
| PLAT | 1.704 | ↑ | 1.605 | ↑ |  |  |  |  | 1.377 | ↑ | -1.933 | ↓ |
| PLCG2 | 1.352 | ↔ | 1.239 | ↔ |  |  |  |  | 1.122 | ↔ | -1.47 | ↔ |
| PPARG | -0.877 | ↑ |  |  |  |  |  |  |  |  | 0.982 | ↓ |
| PRF1 | 2.173 | ↓ |  |  |  |  |  |  | 1.263 | ↓ | -1.632 | ↑ |
| PROS1 | 0.861 | ↑ |  |  |  |  |  |  |  |  |  |  |
| PTGER3 | -1.199 | ↓ | -1.027 | ↓ |  |  |  |  |  |  | 1.282 | ↑ |
| PTGS1 | 1.405 | ↑ | 1.341 | ↑ |  |  |  |  | 1.386 | ↑ | -1.36 | ↓ |
| PTGS2 | 2.837 | ↑ | 1.716 | ↑ |  |  |  |  | 2.194 | ↑ | -2.36 | ↓ |
| PTPN6 | 1.627 | ↔ | 1.528 | ↔ |  |  |  |  | 1.184 | ↔ | -1.97 | ↔ |
| RARRES2 | -0.833 | ↑ | -1.158 | ↑ |  |  |  |  |  |  |  |  |
| RORA |  |  |  |  |  |  |  |  | 1.284 | ↔ |  |  |
| S100A9 | 1.295 | ↓ | 1.316 | ↓ |  |  |  |  | 1.72 | ↓ |  |  |
| SBNO2 |  |  |  |  | 1.202 | ↔ |  |  |  |  |  |  |
| SERPINF1 |  |  | -0.909 | ↓ |  |  |  |  |  |  |  |  |
| SLC11A1 |  |  | 1.338 | ↑ |  |  |  |  | 1.525 | ↑ |  |  |
| SOCS1 |  |  |  |  | 1.305 | ↔ |  |  |  |  |  |  |
| STAT1 | 1.518 | ↑ |  |  |  |  | 1.41 | ↑ | 1.565 | ↑ |  |  |
| STAT4 | 1.883 | ↑ | 1.454 | ↑ |  |  |  |  | 2.435 | ↑ |  |  |
| SYK | 1.979 | ↔ | 1.357 | ↔ |  |  |  |  | 1.462 | ↔ | -1.874 | ↔ |
| TGM2 | 1.334 | ↔ | 1.126 | ↔ |  |  |  |  | 1.51 | ↔ |  |  |
| THBS1 |  |  |  |  | 2.064 | ↑ |  |  | 1.897 | ↑ |  |  |
| TIMD4 |  |  | 3.876 | ↓ |  |  |  |  | 4.449 | ↓ |  |  |
| TLR2 |  |  | 1.253 | ↑ |  |  |  |  | 1.687 | ↑ |  |  |
| TLR6 |  |  | 1.784 | ↑ |  |  |  |  |  |  | -2.176 | ↓ |
| TMEM106A | 1.113 | ↔ |  |  |  |  |  |  |  |  |  |  |
| TRPM2 | 1.758 | ↑ |  |  |  |  |  |  |  |  |  |  |
| TYROBP | 1.273 | ↔ |  |  |  |  | 0.959 | ↔ | 1.334 | ↔ |  |  |
| UNC13D | 1.567 | ↔ |  |  |  |  |  |  | 1.487 | ↔ | -1.222 | ↔ |
| WNT5A | 2.773 | ↑ | 1.599 | ↑ | -1.402 | ↓ |  |  | 1.372 | ↑ | -3 | ↓ |

FC = Log Fold-change, P = Predicted affect, **↑** = Increased, ↓ = Decreased, **↔** = Affected

S2- **Characterization of MSCs and EVs;**


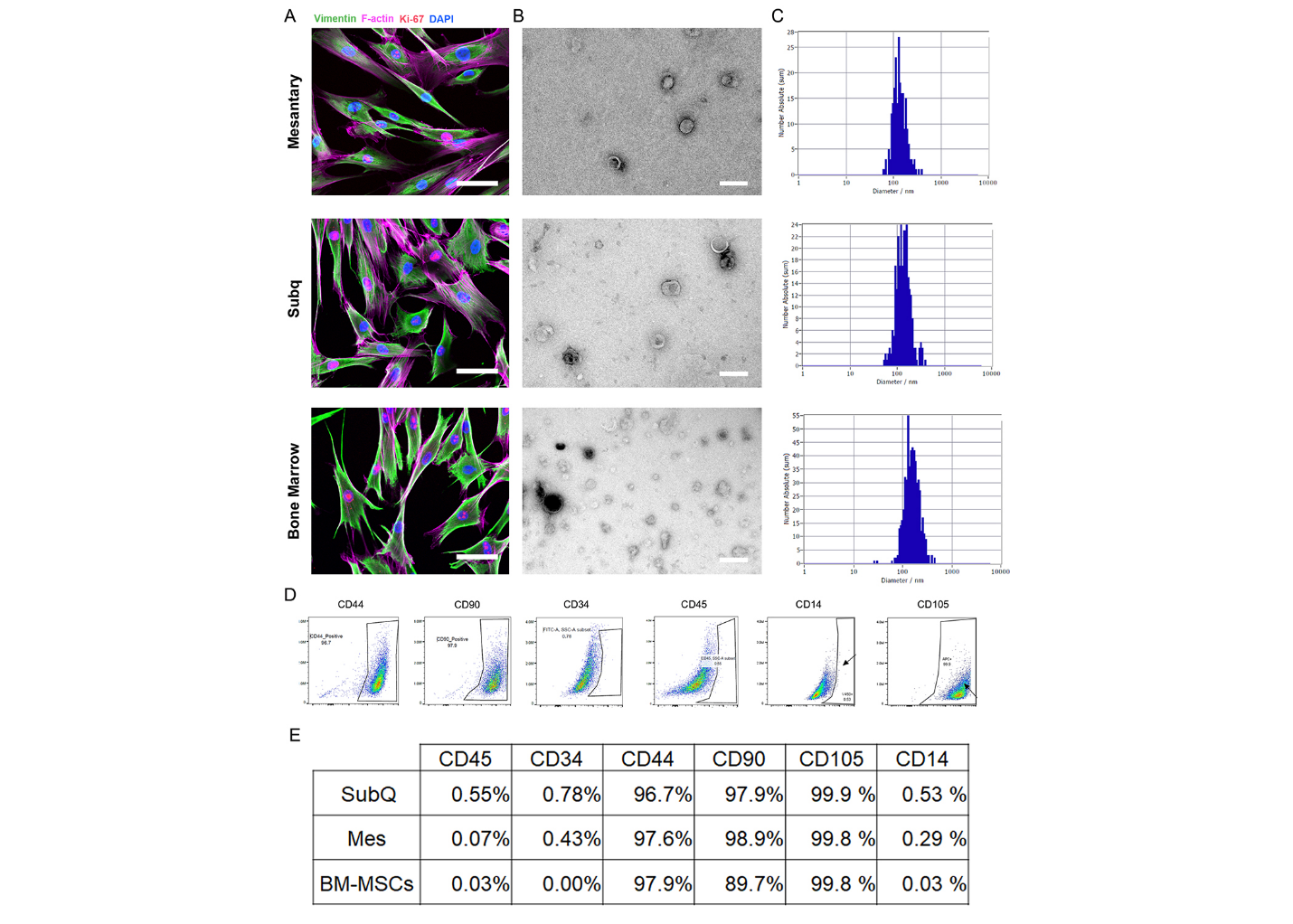


Figure S2- A) Mesentery and Subq cell stained with vimentin ,F-actin and ki-67 compared to Bone marrow derived MSCs, B) TEM images of isolated EVs of MSCs, D)Size scattered of isolated EVs , D and E) CD markers of MSCs for characterization of them
